# Draft Genome Sequence of Streptococcus suis Strain SsRC-1, a Human Isolate from a Fatal Case of Toxic Shock Syndrome

**DOI:** 10.1128/genomeA.00447-18

**Published:** 2018-05-17

**Authors:** Silvia Fillo, Fabiola Mancini, Anna Anselmo, Antonella Fortunato, Giovanni Rezza, Florigio Lista, Alessandra Ciervo

**Affiliations:** a Scientific Department, Army Medical Center, Rome, Italy; b Department of Infectious Diseases, Istituto Superiore di Sanità, Rome, Italy

## Abstract

Streptococcus suis is an economically important pathogen in the pig industry and is also an emerging zoonotic agent responsible for severe infections in humans. Here, we report the genome sequence of S. suis strain SsRC-1. Specifically, this strain was a serotype 2 and was isolated from a human fatal case of toxic shock syndrome (TSS) in Italy.

## GENOME ANNOUNCEMENT

Streptococcus suis is a swine pathogen important in the pig industry economy. This microorganism is also an emerging human pathogen that affects people who are exposed to infected pigs or who handle raw pork. S. suis infection is typically an occupational disease, but several predisposing factors, such as splenectomy or malignancy, may favor the infection (1, 2). Of the 35 identified serotypes, only a few are accountable for human infection, which are usually assigned to serotype 2, sequence type 1 (ST1) (1).

Here, we report the draft genome sequence of the S. suis SsRC-1 strain, isolated from a 55-year-old man who lived and worked on a farm in South Italy (3). The patient, with a previous splenectomy for a Hodgkin’s lymphoma, presented with severe sepsis, multiple organ failure, and disseminated intravascular coagulopathy. The patient died 28 days after hospital admission. The strain was isolated from blood culture, and the bacterium identification was conducted by microbiological and molecular methods. Specifically, this strain was serotype 2, belonged to sequence type 1, and exhibited the following virulence and antimicrobial resistance gene profiles: *mrp*^+^/*epf^+^/sly*^+^/*arc*A^+^/*ofs^type1^/cps2*, *erm*(B), *tet*(O/W/32/O), and *tet*(40) (3).

For genome sequencing, the microorganism was grown in LB liquid culture, and whole DNA was extracted using a MasterPure Gram-positive purification kit (Lucigen Co., WI). The library was prepared using a Nextera XT DNA sample preparation kit v3 (Illumina, San Diego, CA), and a 2 × 300-nucleotide (nt) paired-end sequencing run was performed on an Illumina MiSeq platform. The reads were trimmed and *de novo* assembled using the SPAdes genome assembler (4). The assembly of reads generated 58 contigs of >500 nt, with a 213× coverage. The estimated genome size is 2,093,539 bp, with a G+C content of 42.0%, and there are 2,100 coding sequences (CDSs) that account for 97.44% of the genome (2,155 genes), 45 tRNAs, 6 rRNAs, 4 noncoding rRNAs, and 73 pseudogenes. The genome of the S. suis SsRC-1 strain shows some candidate virulence genes, such as *pgdA*, *dltA*, *ccpA, endoD*, *gtfA*, *purA*, *scrB*, *cdd*, and *neuC* (5). The S. suis SsRC-1 strain was similar to three Italian strains, namely, two clinical isolates, SsCA-1 and SsUD, and S. suis strain 32457, which was isolated from a pig (6–8). This draft genome sequence will offer insight about the genetics of S. suis and will be helpful for comparing strains from different geographic sites.

### Accession number(s).

This whole-genome shotgun project has been deposited in DDBJ/ENA/GenBank under the accession number PYUF00000000. The version described in this paper is version PYUF01000000.

## References

[1] Lun ZR, Wang QP, Chen XG, Li AX, Zhu XQ 2007 *Streptococcus suis*: an emerging zoonotic pathogen. Lancet Infect Dis 7:201–209. doi:10.1016/S1473-3099(07)70001-4. 17317601

[2] Gottschalk M, Xu J, Calzas C, Segura M 2010 *Streptococcus suis*: a new emerging or an old neglected zoonotic pathogen? Future Microbiol 5:371–391. doi:10.2217/fmb.10.2. 20210549

[3] Mancini F, Adamo F, Creti R, Monaco M, Alfarone G, Pantosti A, Ciervo A 2016 A fatal case of streptococcal toxic shock syndrome caused by *Streptococcus suis* carrying *tet* (40) and *tet* (O/W/32/O), Italy. J Infect Chemother 22:774–776. doi:10.1016/j.jiac.2016.05.011. 27553071

[4] Bankevich A, Nurk S, Antipov D, Gurevich AA, Dvorkin M, Kulikov AS, Lesin VM, Nikolenko SI, Pham S, Prjibelski AD, Pyshkin AV, Sirotkin AV, Vyahhi N, Tesler G, Alekseyev MA, Pevzner PA 2012 SPAdes: a new genome assembly algorithm and its applications to single-cell sequencing. J Comput Biol 19:455–477. doi:10.1089/cmb.2012.0021. 22506599 PMC3342519

[5] Feng Y, Zhang H, Wu Z, Wang S, Cao M, Hu D, Wang C 2014 *Streptococcus suis* infection: an emerging/reemerging challenge of bacterial infectious diseases? Virulence 5:477–497. doi:10.4161/viru.28595. 24667807 PMC4063810

[6] Camporese A, Tizianel G, Bruschetta G, Cruciatti B, Pomes A 2007 Human meningitis caused by *Streptococcus suis*: the first case report from north-eastern Italy. Infez Med 15:111–114. 17598998

[7] Manzin A, Palmieri C, Serra C, Saddi B, Princivalli MS, Loi G, Angioni G, Tiddia F, Varaldo PE, Facinelli B 2008 *Streptococcus suis* meningitis without evidence of animal contact, Italy. Emerg Infect Dis 14:1946–1948. doi:10.3201/eid1412.080679. 19046529 PMC2634631

[8] Palmieri C, Magi G, Mingoia M, Bagnarelli P, Ripa S, Varaldo PE, Facinelli B 2012 Characterization of a *Streptococcus suis tet*(O/W/32/O)-carrying element transferable to major streptococcal pathogens. Antimicrob Agents Chemother 56:4697–4702. doi:10.1128/AAC.00629-12. 22710115 PMC3421841

